# Straightforward Magnetic Resonance Temperature Measurements Combined with High Frame Rate and Magnetic Susceptibility Correction

**DOI:** 10.3390/bioengineering10111299

**Published:** 2023-11-09

**Authors:** Sangwoo Kim, Donghyuk Kim, Sukhoon Oh

**Affiliations:** 1 Department of Radiological Science, Daewon University College, Jecheon 27135, Republic of Korea; radmri@daewon.ac.kr; 2 Neuroscience Research Institute, Gachon University, Incheon 21988, Republic of Korea; dhkim04@gachon.ac.kr; 3 Center for Research Equipment, Korea Basic Science Institute, Cheongju 28119, Republic of Korea

**Keywords:** temperature reading, susceptibility correction, fast temporal resolution, proton resonance frequency shift, hyperthermia

## Abstract

Proton resonance frequency shift (PRFS) is an MRI-based simple temperature mapping method that exhibits higher spatial and temporal resolution than temperature mapping methods based on T1 relaxation time and diffusion. PRFS temperature measurements are validated against fiber-optic thermal sensors (FOSs). However, the use of FOSs may introduce temperature errors, leading to both underestimation and overestimation of PRFS measurements, primarily due to material susceptibility changes caused by the thermal sensors. In this study, we demonstrated susceptibility-corrected PRFS (scPRFS) with a high frame rate and accuracy for suitably distributed temperatures. A single-echo-based background removal technique was employed for phase variation correction, primarily owing to magnetic susceptibility, which enabled fast temperature mapping. The scPRFS was used to validate the temperature fidelity by comparing the temperatures of fiber-optic sensors and conventional PRFS through phantom-mimicked human and ex vivo experiments. This study demonstrates that scPRFS measurements in agar-gel are in good agreement with the thermal sensor readings, with a root mean square error (RMSE) of 0.33–0.36 °C in the phantom model and 0.12–0.16 °C in the ex vivo experiment. These results highlight the potential of scPRFS for precise thermal monitoring and ablation in both low- and high-temperature non-invasive therapies.

## 1. Introduction

Thermal treatments, such as thermo-ablation above 50 °C and hyperthermia at 40–45 °C, are widely employed for the clinical treatment of cancer in various parts of the body, including the breast, liver, and prostate [1,2,3]. Regional hyperthermia (RHT), in particular, serves as a viable alternative to radiotherapy or chemotherapy for treating intermediate-phase tumors [4,5]. According to a literature review, tumors with characteristics like hypoxia, poor nutrition, and low pH, which often resist radiotherapy, respond favorably to heat-induced cell death [6]. However, ensuring temperature precision is crucial for the success of RHT as a clinical therapy for cancer [7]. Maintaining a homogeneous temperature of up to 43 °C for 30–60 min within the target cancer area poses a significant challenge for RHT [8,9]. The difficulty arises from the inherent physical and physiological properties of tissues, which lead to variations in the heating effect over time and space [6,10]. To achieve high-quality temperature monitoring in this context, the use of fiber-optic temperature sensors (FOSs) is essential for minimally invasive thermotherapy. Nevertheless, the limited number and placement of probes cannot guarantee temperature accuracy for RHT, particularly when heating a large volume, as the overall temperature distribution becomes less representative [6,7]. This underscores the need for appropriate methods to accurately monitor the heating effect in the RHT process.

Thermometry based on magnetic resonance (MR) enables the simultaneous placement of fiber-optic probes for anatomical reference and non-invasive temperature readings [1,11]. Proton resonance frequency shift (PRFS) is a commonly used method due to its superior spatial and temporal resolution compared to alternatives such as T1 relaxation time- and diffusion-based temperature mapping [1,3,11,12]. However, a significant challenge in PRFS temperature monitoring is the presence of background phase variations, primarily caused by intense gradient switching that heats the main magnetic field [13] and requires compensation for accuracy [14]. A viable solution involves positioning oil phantoms, which induce fewer phase shifts than the background phase variation, around the subject to estimate the background field variation. However, this method can be somewhat cumbersome [15]. Furthermore, addressing the following challenges is essential: phase delays in MR images resulting from electrical shading effects due to temperature changes when heating large volumes and phase variations caused by material susceptibilities, such as implant insertion [16,17]. The phase delay can be resolved using a dual-echo PRFS technique [1]. However, to ensure precise temperature monitoring during PRFS, it is crucial to compensate for phase variations arising from the surrounding macroscopic fields with material properties. Several studies have indicated that factors, like the specific orientation of a needle applicator for ablation [17,18], and material susceptibility, such as coronary stents, can lead to both underestimation and overestimation of temperature in PRFS [19,20]. In essence, varying magnetic susceptibilities within a magnetic field during PRFS can result in diverse temperature errors.

Several studies have attempted to address the impact of magnetic susceptibility (∆χ) on PRFS sensitivity. A susceptibility-induced phase shift proximal to a cryoablation ice ball can lead to a severe temperature error of approximately 10–12 °C, which is corrected by applying a rapid numerical algorithm that estimates magnetic field perturbation [21]. Moreover, this identical algorithm has successfully rectified the temperature error caused by magnetic susceptibility in gas/carbonized tissues within a microwave ablation zone [22]. Additionally, applying background field removal and quantitative susceptibility mapping to the original phase images in the PRFS process enables the elimination of magnetic susceptibility effects due to motion-related artifacts and background phase drifts [23,24]. This approach has been shown to yield temperatures with an acceptable error range when compared to FOSs. An analysis using root mean square error (RMSE) for a phantom and thigh experiment in a region with minimal motion showed differences of approximately 0.21–0.88 °C, which can be attributed to temperature errors arising from the magnetic susceptibility of the FOS materials. The PRFS method itself has a fidelity of ±1 °C [25], but this may include the magnetic susceptibility error caused by the FOS material. MR-compatible FOSs are typically made of materials like glass or polymers, with polymeric materials exhibiting diamagnetic properties and weak phase transitions within the applied magnetic field [26]. It is plausible that FOSs can introduce magnetic field changes due to the material’s susceptibility, leading to temperature errors during PRFS. Considering that magnetic susceptibility perturbations induced by FOSs can be corrected, PRFS has the potential to become a robust and precise tool for non-invasive temperature imaging in efficient RHT procedures.

A literature review has revealed that heating at least 90% of a given tumor volume to a minimum of 43 °C for a cumulative time of at least 10 min can double the response and duration of response to hyperthermia and radiotherapy compared to radiotherapy alone [6]. Since tumor damage depends on both temperature and exposure time, which contribute to cell death, precise temperature monitoring with high spatial and temporal resolution is crucial for assessing the tumor region affected by thermal energy [27]. The authors have emphasized that achieving temporal resolution of less than a second allows for more accurate treatment procedures, as it enables simultaneous monitoring of temperature gradients and spatial resolution. In this study, we employed a background removal technique based on a single echo that incorporates improved harmonic phase removal using the Laplacian operator (iHARPERELLA) [28], as several multi-echo approaches can introduce acquisition delays [29]. This step is performed before the heating process to facilitate rapid temperature mapping, as the location of the optic probes remains fixed during the temperature rise. Our goal was to enhance temperature accuracy by correcting for the material susceptibility of fiber-optic probes. Furthermore, we aimed to achieve superior temperature mapping at a high frame rate through a straightforward real-time approach for monitoring temperature changes during regional hyperthermia therapy (RHT). This study demonstrates that the exceptional PRFS technique, when combined with high-frame-rate capabilities and susceptibility correction, holds the promise of non-invasive thermotherapy with accurate temperature measurements for hyperthermia therapy.

## 2. Materials and Methods

### 2.1. Calculation of the Susceptibility-Corrected Value of PRFS

A phantom was prepared to observe the temperature distributions for the susceptibility-corrected PRFS (scPRFS) relative to the original PRFS (oPRFS). To mimic the average electrical properties of human tissues, that is, the electrical conductivity and relative permittivity, a solution containing agar (8 g/L), NaCl (10 g/L), and CuSO_4_ (1 g/L) was prepared and diluted in hot water. The agar solution was subsequently solidified in a 106 × 74 × 90 mm^3^ plastic container and cooled to room temperature. The phantom was placed inside the MRI machine for more than 24 h to achieve temperature equilibrium before the PRFS experiments [30,31].

**Figure 1:**
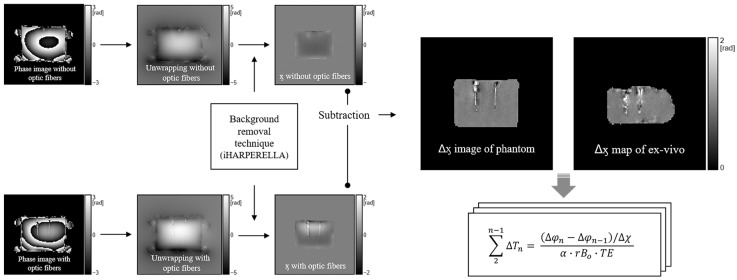
Extraction of susceptibility difference map. The original phase map was unwrapped and subsequently processed using the background removal technique to obtain the magnetic susceptibility difference map (∆χ) by subtracting the susceptibility values from each phantom with and without fiber-optic probes. The application of the ∆ӽ map to the PRFS process (∆φ) caused a phase shift resulting from the correction of fiber optics. In addition, the generation and application of the Δӽ map for an ex vivo experiment involve the same process as for the phantom. iHARPERELLA = improved harmonic phase removal using the Laplacian operator; PRFS = proton resonance frequency shift.

Whole-body 3 T MRI (Achieva, Philips, Best, Netherlands) with a 32-channel phased-array head coil (SENSE Head coil 3.0 T, Philips, Best, Netherlands) was employed to acquire the susceptibility-corrected value for PRFS and temperature readings during the PRFS. To ensure no substantial change in the placement of phantoms after heating in a microwave oven, the phantom was placed on a flat acrylic plate, and four oil phantoms were placed around it. We first implemented 3D-GRE imaging to determine the volume susceptibility of the phantom without heating and FOS insertion (Figure 1). The 3D-GRE with identical parameters and conditions was subsequently repeated with the insertion of an FOS into the phantom. The FOS system comprised a multi-channel signal conditioner with eight channels (AccuSens; Opsens, Quebec, Canada) and temperature sensors (OTP-M; Opsens, Quebec, Canada). These sensors are compatible with EM/RF/MR/microwave fields and possess an accuracy of ±0.3 °C, a 1.2 mm diameter, a fast response time of <1 s, and a temperature-sensing crystal housed in a polyvinyl chloride tip. A 4 cm needle with a 1.5 mm diameter was inserted into the phantom to establish the FOS position. Subsequently, after removing the catheter, the FOS was reinserted into the vacant space to estimate magnetic susceptibility. The scan parameters were repetition time (TR) = 16 ms, echo time (*TE*) = 10 ms, echo number = 1, flip angle = 15°, field of view = 200 × 200 × 100 mm^3^, matrix size = 100 × 100, slice thickness = 5 mm, slice numbers = 20, bandwidth = 2302.6 Hz/pixel, number of averages = 2, and acquisition time = 26 s. The magnitude and phase images that were acquired in the two aforementioned cases were reconstructed using the background removal technique in STI Suite packages [28] to obtain the phase changes induced by volume susceptibilities with the material properties of the phantom and FOS. Mask images for background removal were generated using the Brain Extraction Tool (BET) algorithm in FSL (FMRIB, University of Oxford, Oxford, UK) [32]. Subsequently, the susceptibility-corrected values, $∆\chi_{n}$, were calculated by subtracting the processed volume susceptibility with and without using the fiber-optic probes (Figure 1), as follows.

(1) $$∆\chi =\chi^{with}-\chi^{without},$$

where χ is the volume susceptibility with and without the FOS. When applying the ∆*χ* map to PRFS, a constant of 1 was incorporated in the ∆χ map to prevent the temperature from increasing in areas with no difference between the presence and absence of the fiber-optic sensor and to compensate only for the difference in the magnetic susceptibility.

### 2.2. Phantom and Ex Vivo Experiment for Temperature Reading of PRFS

The phantom was used again to observe tendencies of temperature change corresponding to an approximately 6 °C increase in the RHT. A microwave oven with 700 W nominal output (RE-C21VW, Samsung, Republic of Korea) was used to heat the phantom for 2 min outside the MR room. The heated phantom reverted to the MR room and was placed as close as possible to its previous position on the acrylic plate. In addition, we inserted the FOS into the phantom at its established location before the heating and monitored the temperature change. To measure the temperature changes based on the PRFS techniques, the phantom was scanned using an identical MRI scanner with a phased-array RF coil. We employed an EPI sequence for high-frame-rate temperature mapping, which was similar to the 3D-GRE mentioned above. The parameters selected were as follows: repetition time (TR) = 21 ms, echo number = 2, echo time (*TE*) = 5/15 ms, flip angle = 30°, field of view = 200 × 200 mm^2^, matrix size = 100 × 100, slice thickness = 5 mm, slice number = 1, bandwidth = 281.9 Hz/pixel, number of averages = 2, EPI factor = 5, and acquisition time = 0.8 s. The EPI scan protocol was continuously repeated to monitor temperature alterations during 600 s. The magnitude and phase images acquired through EPI were exported for temperature mapping per scan. Phase retardation induced by the electromagnetic properties of tissues occurs mainly in the case of wide-area heating that is simply corrected with a phase image of ∆*TE* extracted from the dual-echo images [1,11,25]. Thus, we applied a complex conjugate process at the first and second *TE*s to obtain a phase image with ∆*TE* [33,34], as follows.

(2) $$S_{\Delta TE}=S_{TE2}\cdot S_{TE1}^{*}$$

where *S* denotes the complex data of MR images, $S_{TE1}^{*}$ is the complex conjugate, and ∆*TE* is the difference value (10 ms, *TE*2−*TE*1). Subsequently, the phase images in all *TE*s were calculated using the cumulative PRFS process for the oPRFS, as follows:

(3) $$∆T_{n}=\sum_{i=2}^{m}\frac{\left(\varphi_{\left(i,n\right)}-\varphi_{\left(i-1,n\right)}\right)-{∆\varphi }_{f,n}}{\alpha \cdot \gamma B_{o}\cdot {TE}_{n}}\;$$

where α is the PRFS coefficient (0.01 ppm/°C), $B_{o}$ is the main magnetic field strength, *m* is the total scan number during 600 s, *n* is the value of *TE*s (set to 5, 10, and 15 ms), φ is the phase images with each *TE* (5 and 15 ms) obtained using EPI and with the ∆*TE* (10 ms) obtained from Equation (2), and ${∆\varphi }_{f}$ is a background field drift map. First, the EPI phase images with the *TE*1 acquired over 600 s were subtracted from each other, as expressed in Equation (3). Four oil phantoms that were already placed around the phantom were used to compensate for the background field drifts caused by eddy current effects [11,14,15,30,31,35]. After subtracting the phase images, the signals within each oil phantom were averaged to produce a single value. The four averaged values on each processed phase image were used to create the field drift map (${∆\varphi }_{f,n}$) through two-dimensional linear interpolation and extrapolation with a first-order polynomial curve fitting. The field drift correction was applied using the drift map to adjust the subtracted phase image. Furthermore, the phase images with *TE*2 and ∆*TE* executed the cumulative PRFS process in the same manner as with *TE*1, completing the oPRFS. Second, the volume susceptibility differences, ∆χ, were divided to correct the phase changes caused by the material properties of the FOS in the PRFS process. A volume susceptibility slice with the observed FOS was selected and registered to all phase images using FMRIB’s Linear Image Registration Tool (FLIRT [36,37]) to reduce the position error despite a similar phantom position between the ∆*χ* map and all heated phase images. The FLIRT exported registration coordinates between the magnitude images from the EPI with *TE*1 (because shorter *TE* is high SNR) and those in the GRE for the ∆*χ* map; furthermore, the coordinates were used when registering the ∆χ map to the phase images. Finally, the scPRFS temperature procedure was identical to the oPRFS, except that the ∆*χ* map was applied after correcting for the background field drift at each *TE*.

**Figure 2:**
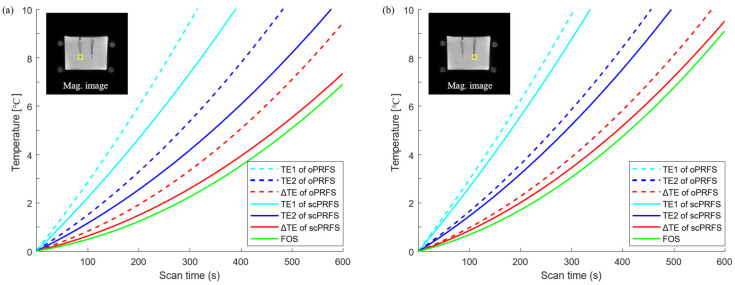
Temperature changes of individual ROIs. (**a**) A scPRFS with Δ*TE* (left yellow box) was increased slightly more than the temperature with a fiber-optic sensor; (**b**) Another ROI (right yellow box) also showed a comparable temperature to that of the fiber-optic sensor. However, the degree of difference and the temperature difference were sufficiently in line with the PRFS criterion. scPRFS = susceptibility-corrected PRFS; Δ*TE* = 10 ms; *TE*1 = 5 ms; *TE*2 = 15 ms; Mag = magnitude.

The temperature changes in both oPRFS and scPRFS were measured by averaging the values within the region of interest (ROI), which consisted of 4 × 4 pixels, including the central position of the FOS (see Figure 2). These measurements were then compared with those obtained from the FOS. All temperatures of the FOS were subtracted from the first temperature to match the temperature expression of the PRFS, thereby displaying the relative temperature changes [3]. Finally, RMSE analysis was performed to compare the temperature difference of all *TE*s in the PRFS methods with that of the FOS.

Additionally, we conducted an ex vivo experiment to validate the scPRFS. The ex vivo experiment involved placing swine thigh muscle tissue in a plastic container of the same size used in the phantom experiment. The muscle tissue was obtained from a local butcher the day before the experiment and stored in a refrigerator at 4 °C. It was then allowed to reach room temperature for 8 h before the experiment began to ensure temperature equilibrium with the MRI room. The needle used for the phantom was inserted into and removed from the ex vivo specimen, and the FOS was placed in the same location. Assuming the heating of a large amount of material, one fiber-optic sensor was placed in the center and the remaining in the surrounding area for the RHT. In a human-mimicked phantom, determining the exact temperature is difficult because the center does not contain a fiber optic thermometer. Therefore, we placed one of the fiber-optic sensors at the center and one on the periphery in an ex vivo experiment to confirm the usefulness of scPRFS. The 3D-GRE sequence for exporting the susceptibility value was implemented before and after the inclusion of the FOS with a scan parameter identical to that of the phantom test without the heating effect. In addition, all temperature calculation methods for the oPRFS and scPRFS, such as the heating process, heating time, EPI scan parameters, background field drift correction with oil phantoms, and correction of the susceptibility difference of the FOS, were identical to those employed in the phantom experiment. However, by referring to a previous study, the PRFS coefficient was set to a value of 0.009 ppm/°C for pig muscle [38].

## 3. Results

**Figure 3:**
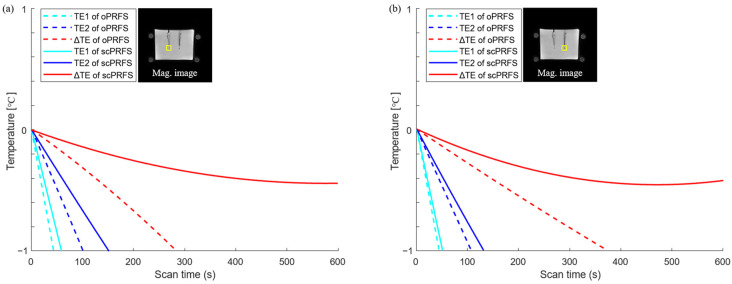
Temperature errors in each position (yellow boxes) with an increase in temperature. Temperature error values were calculated by subtracting each PRFS temperature from the individual heating checkpoint of each fiber-optic temperature. (**a**) A scPRFS with Δ*TE* (red line, left ROI) was comparable to the average temperature of the fiber sensor and sufficiently met the PRFS criterion corresponding to a temperature difference of ±1 °C in all heating durations; (**b**) The temperature error in another ROI (right yellow box) showed the similar tolerance. oPRFS = original PRFS; scPRFS = susceptibility-corrected PRFS; Δ*TE* = 10 ms; *TE*1 = 5 ms; *TE*2 = 15 ms; Mag = magnitude.

This study was performed to enhance the accuracy of regional phase-based MR thermometry (PRFS) by compensating for the susceptibility value of fiber optics from the phase images (scPRFS). The scPRFS showed virtually resembling temperature changes from the FOS (Figure 2), particularly for Δ*TE*. In one individual region (Figure 2a and Figure 3a), the scPRFS with Δ*TE* increased by approximately 7.38 °C (FOS = 6.88 °C, RMSE = 0.33 °C). The other region of scPRFS with Δ*TE* exhibited approximately 9.61 °C (Figure 2b, FOS = 9.09 °C) with an RMSE of approximately 0.36 °C (Figure 3b). Except for Δ*TE* within both ROIs, scPRFS with *TE*1 and *TE*2 consistently overestimated the temperature values as the temperature increased (see Table 1). Furthermore, oPRFS temperatures with all *TE*s exhibited greater errors compared to scPRFS. The temperature values for each *TE* and the FOS within each region of the phantom are summarized in Table 1.

**Figure 4:**
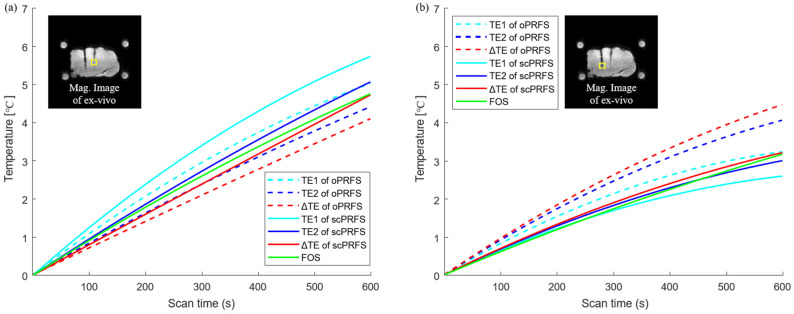
Individual temperature reading for the ex vivo study. Each temperature monitoring of scPRFS was calculated in the center and outer regions (yellow box). (**a**) The central scPRFS values (red line) showed a comparable temperature to that of the optical fibers; (**b**) The temperature in the outer region was increased by lower values relative to the temperature increase in the central region, but also showed a temperature similar to that of the optical fiber. Although the temperature of the scPRFS with *TE*2 is similar to that of the fiber optic sensor, the temperature along each *TE* varies depending on the measurement location.

Furthermore, we observed the temperature changes to validate the impact of scPRFS on the ex vivo state. In the center region, scPRFS with Δ*TE* showed a temperature change of approximately 4.73 °C (RMSE ≈ 0.16 °C), whereas the FOS registered approximately 4.76 °C (see Figure 4a). On the other hand, oPRFS with Δ*TE* underestimated the temperature at approximately 4.10 °C. While oPRFS and scPRFS with *TE*1 and *TE*2 exhibited similar temperature patterns with increasing temperature when compared to the FOS, scPRFS with Δ*TE* displayed the smallest temperature difference from the FOS. In the outer region, scPRFS with Δ*TE* also showed temperatures more comparable to the FOS, measuring approximately 3.02 °C (RMSE ≈ 0.12 °C) compared to approximately 3.17 °C recorded by the FOS (see Figure 4b). Notably, a minimal temperature error was observed in this region as well (refer to Table 1).

## 4. Discussion

This study was designed to obtain a high frame rate and accuracy for the oil-based PRFS technique by correcting the susceptibility values of fiber-optic probes. We demonstrated that, compared with fiber optics, scPRFS with Δ*TE* (RMSE = 0.33–0.36 °C) exhibited substantial agreement with fiber-optic temperature measurements in both the phantom (Figure 2) and ex vivo studies (RMSE = 0.12–0.16 °C, Figure 4). The susceptibility-corrected PRFS technique not only improves the sensitivity of temperature monitoring without having to adjust the temperature-dependent constant factor α but can also be utilized for achieving accurate thermal ablation in interstitial laser-induced thermal therapy using fiber-optic probes.

We found that the temperatures of scPRFS with Δ*TE* were identical to those of fiber optics, suggesting that this method can simultaneously correct temperature errors caused by magnetic susceptibility and phase retardation. The magnetic susceptibility of fiber optics directly affects the temperature sensitivity owing to the phase shift from micro- and macroscopic perspectives [27,39,40,41]. Previous studies on PRFS have revealed that electrically conductive implants cause temperature errors that increase owing to excess electromagnetic induction [19,20,21,22]. A prior study has demonstrated approximately 5–16 °C errors with the position and angle of various RF applicators to the B0-field; these errors cause different temperature shifts owing to the Larmor frequency changes according to material susceptibilities [17]. This is an overestimated result of this study (Figure 2), and it supports the hypothesis that temperature errors are induced by the magnetic properties of fiber-optic probes. Furthermore, prior research that employed various background removal techniques and quantitative susceptibility mapping documented temperature differences of 0.21–0.88 °C compared to measurements obtained through fiber optics in both phantom and in vivo tests [23,24]. This study demonstrated a smaller root mean square error (RMSE) of approximately 0.12–0.36 °C (see Figure 2 and Figure 4) compared to a previous study, indicating the successful removal of the magnetic susceptibility effects associated with fiber optics from the original phase data. This correction allows for accurate temperature monitoring and suggests that scPRFS measures temperature changes solely through the phase shift caused by heating effects.

While temperature monitoring after microwave heating may represent a cooling phenomenon, in this study, we observed an increase in temperature in the phantom and the ex vivo experiments (Figure 2 and Figure 4). This may be a result of the thermodynamic effect on water molecules. PRFS relies on the state of hydrogen bonding between protons and the surrounding oxygen atoms. As the temperature increases, the hydrogen bonds within water molecules stretch, bend, and break, causing water molecules to become more densely packed [42]. This results in increased electron shielding (contributing to a more diamagnetic component), leading to reduced magnetic field strength, lower proton resonance frequency, and a phase shift in aqueous tissues [42,43]. Water molecules are widely recognized as substantial thermal reservoirs, characterized by their high heat capacity and relatively low thermal diffusivity [44]. Low thermal diffusivity indicates that substances have a greater capacity to store heat rather than rapidly dissipate it [45,46]. In this study, the temperature of the phantom (Figure 2) and the ex vivo experiment (Figure 4) gradually increased after microwave heating. This suggests that the rise in the number of water molecules due to microwave heating reduces the thermal diffusivity of the materials. Consequently, this increased ability to store heat and diminished heat dissipation leads to temperature elevation after microwave heating. In addition, for the pig muscle tissues, a PRFS coefficient (α) of 0.009 ppm/°C has been established [38]. This coefficient represents the constant of electronic shielding that changes with temperature and is comparable to that of aqueous tissues, which is approximately 0.01 ppm/°C. This similarity suggests that the porcine muscle tissue has a high water content and lends support to our findings, thus indicating that the temperature patterns observed after microwave heating in this study exhibit a similar increasing trend between the agar phantom and the muscle tissues. Essentially, both the phantom and the muscle tissues demonstrate similar electrical screening effects with temperature variations, thus leading to the gradual temperature changes observed, primarily due to the low thermal diffusivity of hydrogen after heating.

Phase retardation induced by the electromagnetic properties of tissues mainly occurs in cases of a wide area of heating, and it is simply corrected by the dual-echo PRFS method [1,11]. The temperature of scPRFS with Δ*TE* exhibits superior accuracy compared with the other *TE*s that exhibit certain differences during dynamic scans. The severe temperature errors of fiber optics are found for single *TE* at 5 and 15 ms in this study, and the gaps increase with the temperature (Figure 3). This observation suggests that scPRFS with a single *TE* retains the phase retardation accumulated as the degree of phase retardation intensifies owing to decreased relative permittivity and increased electrical conductivity at high-temperature ranges [47,48]. Essentially, the temperature changes in scPRFS with Δ*TE* are generally unaffected by phase retardation at high heating levels, particularly in our study where a large heating area was employed. The accuracy of PRFS temperature monitoring depends on the *TE* selection. In addition, Winter et al. have affirmed that dual-echo PRFS can remove phase retardation due to temperature changes [1]; this strongly supports our findings. It can also be anticipated that laser-induced thermal therapy, particularly when dealing with rapidly elevated temperatures over a short duration using a radiofrequency applicator and fiber-optic probes for heating [49,50], would demand the utilization of scPRFS with Δ*TE* to ensure precise thermal ablation devoid of temperature errors attributed to phase retardation.

This study also accomplishes a high-frame-rate temperature mapping of approximately 0.8 s per slice, implying that scPRFS with Δ*TE* based on an EPI sequence is suitable for dynamic temperature monitoring. Real-time temperature reading is important to ensure accurate therapy by preventing mismatches between the treatment source and target region caused by random or respiratory motion [51] and observing accurate heating levels distributed in massive target regions [7]. Rapid monitoring can also protect normal tissues from high-energy ablations because diseased tissues differ in terms of temperature rise [12,52,53]. Non-Cartesian PRFS has demonstrated the efficiency of a real-time temperature reading with a scan time of less than 1 s in each abdomen slice without motion-related artifacts [54]. In this study, scPRFS with Δ*TE* based on the high-frame EPI sequence exhibited a similar acquisition time to non-Cartesian temperature mapping, and the temperature values in all regions of the ex vivo region were comparable to those of fiber optics after the microwave heating process (Figure 4). This indicates that the center and boundary temperatures for the RHT are accurately measured, and may enable accurate thermo-ablation by targeting disease and protecting normal tissues. Although additional studies are required to verify the treatment fidelity of various cancers and anatomical regions in vivo, high-speed scPRFS with Δ*TE* can decrease temperature errors induced by equipment used during hyperthermia treatment. Consequently, high-frame scPRFS with Δ*TE* could be established as a non-invasive treatment technique with accurate temperature monitoring. Furthermore, scPRFS with Δ*TE*, in conjunction with real-time and precise temperature measurements, is promising for improving RHT sensitivity by ensuring a thorough threshold value is maintained throughout the entire treatment area.

This study has several limitations, which must be addressed in the future. Herein, the susceptibility value of the fiber-optic sensors was extracted before the heating process, and this may induce errors in thermal estimations if the slice position is changed owing to the movement of the subject during the examination. However, it may be resolved based on a new susceptibility value obtained through a 3D-GRE scan, albeit at the cost of a small increase in the acquisition time. In addition, high-frame scPRFS with Δ*TE* should be validated in motion-related regions, such as the liver and pelvis, by a trigger gating for substituting non-Cartesian PRFS [12,54]. Furthermore, the accuracy of PRFS is sensitive to the number of adipose tissues included because lipids do not generate any phase shift [1]. Various methods of separating lipid components have been demonstrated using T1 maps, water, a fat-separated thermal Dixon-based technique, magnetization transfer, T2-relaxation time, and molecular diffusion [1,11]. A comparison of the outcomes of this study with previous results is yet to be conducted. Although scPRFS with Δ*TE* exhibited superior temperature fidelity in a phantom and ex vivo experiment compared with fiber optics, its reproducibility and robustness are yet to be proven through various in vivo studies involving normal and cancerous conditions.

## 5. Conclusions

We implemented a straightforward temperature-mapping technique with a sufficiently high frame rate based on the magnetic susceptibility of the corrected fiber-optic probes. This method suppresses phase variations by considering the magnetic susceptibility and phase retardation for an accurate temperature reading of PRFS. This technique is effective for non-invasive MRI-guided thermo-ablation at low/high temperature ranges for various related diseases, and is expected to enable improved prognosis.

## Figures and Tables

**Table 1:** Temperatures for different *TE* in individual phantom and ex vivo regions, and heated temperatures during a period of 10 min.

Phantom	Individual Region #1		Individual Region #2	
	oPRFS (°C)	scPRFS (°C)	oPRFS (°C)	scPRFS (°C)
*TE*1	21.73	17.08	22.15	20.10
*TE*2	13.50	10.61	14.48	13.11
Δ*TE*	9.38	**7.38**	10.65	**9.61**
Optic fiber [°C]	6.88		9.09	
Ex vivo	Center region		Outer region	
	oPRFS (°C)	scPRFS (°C)	oPRFS (°C)	scPRFS (°C)
*TE*1	5.02	5.73	3.22	2.58
*TE*2	4.41	5.06	4.04	3.00
Δ*TE*	4.10	**4.73**	4.45	**3.20**
Optic fiber (°C)	4.76		3.02	

*TE*1 = 5 ms; *TE*2 = 15 ms; Δ*TE* = 10 ms. Temperatures of scPRFS with Δ*TE* (bold) are closest to those of fiber optics.

## Data Availability

The data presented in this study are available on request from the corresponding author.
